# Massachusetts Veterinary Association

**Published:** 1891-12

**Authors:** 


*Massachusetts Veterinary Association.*—The regular meeting of the Massachusetts Veterinary Association was held at 19 Boylston Place, Boston, Wednesday evening, Oct. 28, 1891, at 7:30 o'clock.

President L. H. Howard in the chair. Members present: Drs. Bryden, Blackwood, Burr, Howard, Hadcock, Marshall, Osgood, Winchester, Winslow and the Secretary. Honorary member: Dr. Stickney. Guest: Dr. E. F. Harrington.

Minutes of last meeting (held in June) read and accepted. Unfinished business. Amending Constitution.

Dr. Osgood thought that the name of anyone proposed for membership should lie over a month before being balloted upon, so as to give ample time for looking up the applicant's record. Dr. Bryden thought that we ought to act in accordance with our State Charter. At the same time if there was any bugaboo it ought to be gotten rid off. The question is upon suspending the clause requiring a thesis as a requirement for admission, for a period of two years from last April. Ballot taken. Resulted in seven votes, all in the affirmative.

The Secretary reported a letter from Dr. Donaldson, of Clark University, acknowledging the receipt of spinal cord of the old pony killed for string-halt, at Ward's Wharf, last summer, and saying that he would report the result of the microscopic examination as soon as it was completed.

The committee consisting of Dr. Winchester and the Secretary, which met a committee from the Board of Control of the State Agricultural Experiment Station, at Amherst, consisting of President Goodell, and Secretary Sessions, of the State Board of Agriculture, Thursday, July 2, at the Commonwealth Building, Boston, to talk over the advisibility of appointing a veterinary pathologist at the State Experiment Station, reported, that the committee that they conferred with were favorably impressed with the idea, and that President Goodell was to formulate a plan of procedure, but that unfortunately he had gone abroad for his health and could do nothing about it at present.

The Secretary reported having seen Mr. Sessions that afternoon, who said that he had not yet given the matter any thought, as he was waiting to see what President Goodell advised doing. Motion made by Dr. Osgood, and seconded by Dr. Winslow, that a vote of thanks be given the committee, and that its report be accepted, and that the committee be continued with full power to act. Carried.

Application for membership received from Dr. E. F. Harrington, and referred to the Executive Committee.

New Business: The Secretary reported the resignation of Dr. A. W. Clement, of Baltimore, who resigned because he had not lived in Massachusetts for three years, and therefore failed to avail himself of the benefits of membership in the Association. Moved by Dr. Winchester, and seconded by Dr. Osgood, that Dr. Clement's resignation be accepted with regrets. Carried.

Dr. Osgood then filed a request in writing, with the Secretary, to still further amend the Constitution by providing that Article III. shall be changed, so that applications for membership in this Association must be made in writing to the Secretary, with the presentation of the applicant's credentials. Said application must be presented to the Association at its next regular meeting, at which time the Secretary is to present the applicant's credentials to the Executive Committee, which after examining the same, shall report to the meeting. If favorably reported on, the name of the applicant shall be laid on the table till the next regular meeting before being voted on. During this time the Secretary shall notify members of the Association in writing of applicant's to be voted upon at each regular meeting. If accepted the Secretary shall notify the applicant of the same.

Reports of cases.

Dr. Stickney reported the case of recovered cœcal fistula in the bay mare killed at Ward's Wharf this afternoon.

A vote of thanks was given Drs. Stickney, Saunders and Peters, for their interest in buying the mare and keeping her until now for post-mortem examination. A number of the members expressed their willingness to contribute their share towards the expense of the same. (A detailed report to be written later, and a copy kept for the Association, and copies given the veterinary journals.)

Dr. Winchester then reported four very interesting cases, as follows: 1. Fistula of Steno's Duct in a horse. 2. Two spleens in a colt, one abnormally large. 3. A case of canine rabies, having a long incubative period. 4. Peritonitis in a horse, following the rupture of the peritoneal coat of the stomach.

Moved by D. Blackwood, seconded by Dr. Marshall, that Dr. Winchester be given a vote of thanks for recording these cases. Carried.

Meeting then adjourned.

AUSTIN PETERS, *Secretary,*

*Ontario Veterinary College, Veterinary Medical Society.*—The Veterinary Medical Society in connection with the Ontario Veterinary College was organized Oct., 21st, with the following office bearers: President, A. Smith, F. R. C. V. S.; Vice-President, C. H. Sweetapple, V. S.; Secretary, J. S. Grove, of Akion, Ohio; Assistant-Secretary, J. W. Watson, Peru, Indiana; Libiarian, F. W. Swearingen, Decatur, Ill.; Treasurer, J. H. Hester, of Nebraska.

This society meets regularly twice a week throughout the session. All the gentlemen of the Senior class being required to read papers before the society, and the discussions on many subjects would do credit to older heads. One of the most interesting meetings thus far was held Wednesday evening, Oct., 28, when the following interesting programme was presented and appropriately discussed:

Essay; S. Somerville, Buffalo, N. Y., "Purpura Hæmorrhagica;" J. J. Elliot, Washington, Ontario, "Enteritis;" Thos. Trinder, Cork, Ireland, "Chorea;" E. L. Button, Durand, Mich., "Alôes."

Communications were made by W. T. Hart, Raveuna, Ohio, "Sitfast;" J. N. Umphery, Udora, Ontario, "Parturient Laminitis;" J. S. Thompson, Galena, Ill., "Nasal Gleet;" D. McCuaig, Bellville, Ontario, "Incised Wound."

J. S. Grove, *Secretary.*

